# *Francisella tularensis*-infected human neutrophils are trojan horses for infection of macrophages

**DOI:** 10.3389/fimmu.2025.1632942

**Published:** 2025-09-04

**Authors:** Sydney M. Escobar, Jenna McCracken, Justin T. Schwartz, Ann M. Miller, Lee-Ann H. Allen

**Affiliations:** ^1^ Department of Molecular Microbiology and Immunology, University of Missouri, Columbia, MO, United States; ^2^ Research Service, Harry S. Truman Memorial VA Hospital, Columbia, MO, United States; ^3^ Department of Microbiology and Immunology, University of Iowa, Iowa City, IA, United States; ^4^ Medical Scientist Training Program, University of Iowa, Iowa City, IA, United States; ^5^ Department of Internal Medicine, University of Iowa, Iowa City, IA, United States; ^6^ Research Service, Iowa City VA Health Care System, Iowa City, IA, United States

**Keywords:** neutrophils, efferocytosis, C1q, macrophage polarization, *Francisella tularensis*

## Abstract

*Francisella tularensis*, the causative agent of tularemia, is a Gram-negative bacterium that infects neutrophils (polymorphonuclear leukocytes, PMNs) and macrophages. Previous studies by our group and others demonstrate that *F. tularensis* inhibits the respiratory burst, escapes the phagosome, replicates in the cytosol, and significantly prolongs human neutrophil lifespan. However, the fate of infected neutrophils and their bacterial cargo are unknown. We now demonstrate that *F. tularensis*-infected neutrophils (iPMNs) interacted more efficiently with primary human monocyte-derived macrophages (MDMs) than aged, control PMNs despite their viability and paucity of surface phosphatidylserine and identified an important role for serum and C1q in this process. Uptake by this mechanism supported bacterial growth in MDMs, indicating that iPMNs can act as Trojan horses to spread infection. Efferocytosis of apoptotic cells favors repolarization of macrophages from a proinflammatory (M1) phenotype to a pro-resolution (M2) phenotype. In marked contrast, the effects of iPMN were distinct, as these cells elicited an atypical MDM phenotype notable for downregulation of both M1 and M2 surface markers that was accompanied by sustained expression of indoleamine 2,3 dioxygenase and suppressor of cytokine signaling 1 as well as low proinflammatory cytokine secretion. Altogether, our data advance understanding of neutrophil-macrophage interactions and reveal a potential new mechanism for *F. tularensis* dissemination and immunomodulation within a host.

## 1 Introduction

The innate immune system serves to identify, contain, and eliminate microbes while also controlling the intensity and duration of the inflammatory response (1). Neutrophils (polymorphonuclear leukocytes, PMNs) are the most abundant leukocytes in humans, migrate rapidly from the bloodstream to sites of infection, and in this locale kill invading microbes via phagocytosis, degranulation and toxic oxidant production. These cells are turned over at a rate of 10^11^ cells/day in humans via a tightly regulated constitutive apoptosis program that is typically accelerated following phagocytosis of bacteria or opsonized particles (2, 3). Efferocytosis is a process whereby macrophages rapidly and efficiently engulf and degrade apoptotic cells that is crucial for two primary reasons (4). First, it prevents accumulation of cellular debris and the release of proinflammatory host and microbe components from dying cells that can damage surrounding tissue or exacerbate autoimmune diseases, such as cystic fibrosis and systemic lupus erythematosus. Second, it reprograms macrophages from a proinflammatory state to an anti-inflammatory phenotype that favors resolution and tissue repair and is characterized by anti-inflammatory cytokine secretion (5). Efferocytosis is rapid, efficient, and associated with accumulation of “eat me” markers such as phosphatidylserine (PS) and downregulation of “don’t eat me” markers such as CD31 and CD47 on apoptotic cell surfaces. Despite PS being well known as the key mediator in efferocytosis in most instances, uptake of apoptotic neutrophils by human monocyte-derived macrophages (MDMs) is generally PS-independent and roles for integrins, complement C1q and mannose-binding lectin (MBL), CD91, and/or calreticulin (CRT) have been defined (6–15).

*Francisella tularensis*, is a Gram-negative, facultative intracellular bacterium that is found throughout the Northern Hemisphere and causes tularemia, a potentially fatal zoonotic disease. Two subspecies of this bacterium, *F. tularensis* subspecies *tularensis* (type A) and *F. tularensis* subspecies *holarctica* (type B), account for nearly all human infections with this organism (16). Humans can acquire tularemia via the bite of an infected arthropod vector, direct contact with an infected animal, ingestion of infected meat or contaminated water, or inhalation of aerosolized bacteria (17, 18)*. F. tularensis* infects and grows within several cell types *in vivo* and *in vitro* including macrophages, neutrophils and epithelial cells (17, 19, 20). Critical to virulence are an atypical LPS that does not signal through Toll-like receptor 4 (TLR4), a surface capsule that confers resistance to complement-mediated lysis, disruption of phagocyte ROS production and phagosome maturation, and a type VI secretion system (T6SS) that is essential for phagosome escape and subsequent bacterial replication in host cell cytosol (21–26).

Particularly relevant here, *F. tularensis* significantly delays neutrophil apoptosis via changes in neutrophil gene expression that alter the abundance of apoptosis regulatory factors, sustain mitochondrial integrity and reprogram metabolism (27–30). It is established that delayed apoptosis is a hallmark of an ineffective and dysregulated immune response that undermines host defense and exacerbates disease (4, 31, 32), and although it is clear that PMN lifespan is prolonged, the fate of infected neutrophils is unknown. Thus, we undertook this study to elucidate the fate of *F. tularensis*-infected neutrophils and their bacterial cargo and hypothesized that diminished apoptosis would undermine infected PMN clearance.

## 2 Results

### 2.1 Interaction of *F. tularensis*-infected PMNs with human macrophages is enhanced

**Figure 1:**
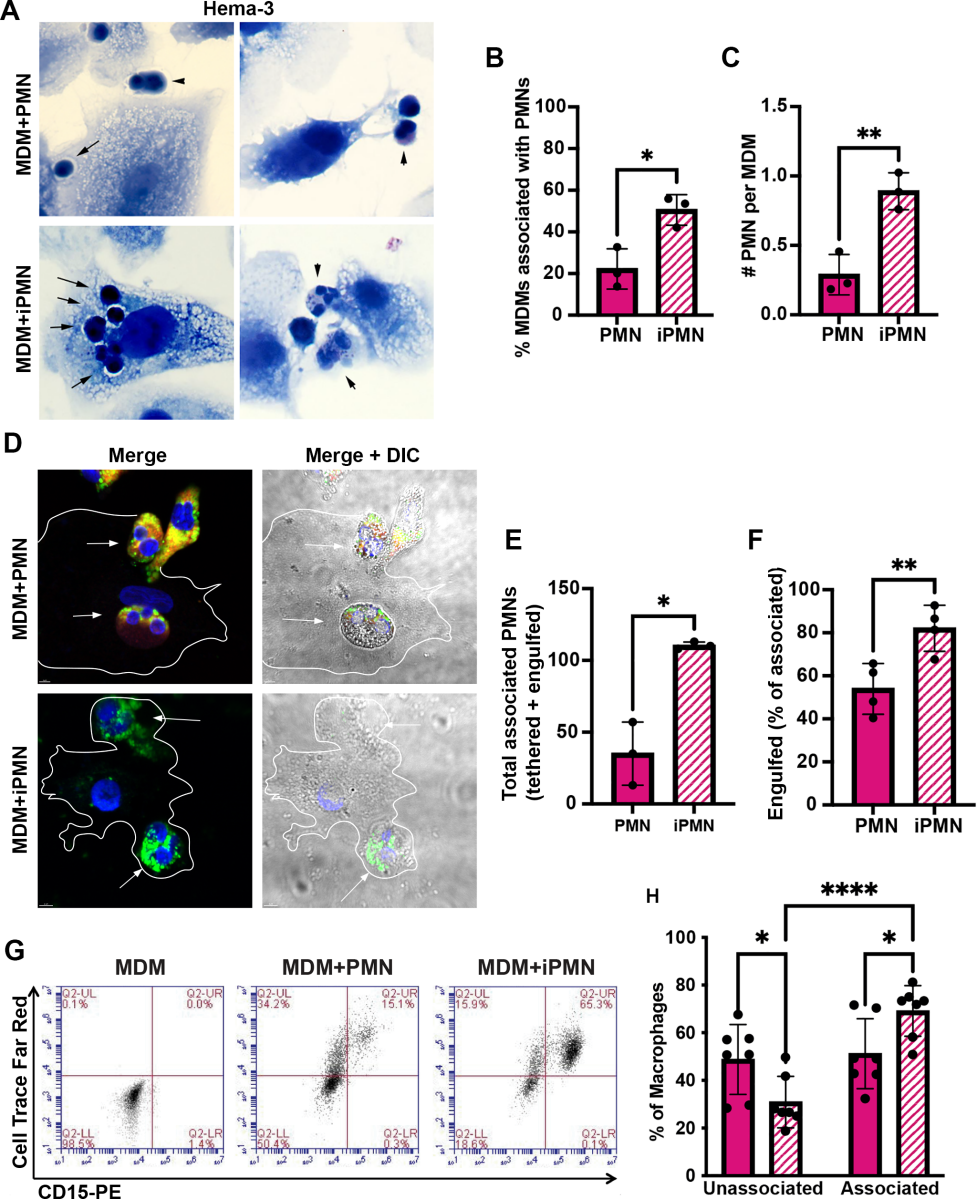
*F. tularensis*-infected neutrophils bind to and are ingested by human macrophages. **(A–C)** Neutrophils were added to monocyte-derived macrophage (MDM) monolayers at a ratio of 5:1 after 24 h of ageing in culture (PMNs) or infection with *F. tularensis* (iPMNs) **(A)** Representative Hema-3-stained images. *Arrows* and *arrowheads* indicate ingested and surface tethered neutrophils, respectively. Pooled data from three independent experiments indicate the percentage of MDMs interacting with PMNs and iPMNs as the mean ± SD (n=3) **(B)** and the total number of neutrophils per interacting macrophage **(C)**. *p<0.05, **p<0.01 by Student’s t-test. **(D–F)** Representative confocal images **(D)** of differentially stained cells show surface-exposed neutrophils as CD15+ (red) and MPO+ (green) whereas fully ingested PMNs are green only. DAPI-stained DNA is in blue. **(E)** Quantitation of total MDM-associated PMNs and iPMNs. Data are the mean + SD (n=4). *p<0.05 by Student’s t-test. **(F)** Quantitation of engulfed PMNs as a percentage of the total MDM-associated Data are the mean + SD (n=4). **p < 0.01 by Student’s t-test. **(G)** Representative flow cytometry plots of MDMs incubated with CellTrace Far Red-labeled aged or infected neutrophils and then stained with anti-CD15-PE. MDMs in the lower left quadrant are not interacting with neutrophils. Cells in the upper left quadrant contain ingested neutrophils only. Cells in the upper right quadrant include MDMs with surface-tethered PMNs or a combination of surface tethered and fully-ingested neutrophils. **(H)** Quantitation of flow cytometry data from 7 independent experiments indicates the percentage of MDMs not interacting with aged or infected PMNs (lower left quadrant) and the percentage of MDMs associated neutrophils (tethered or ingested, sum of upper left and upper right quadrants). *p < 0.05, ****p < 0.0001 by ANOVA with Tukey’s multi-comparison post-test.

Our published data demonstrate that *F. tularensis* significantly delays human neutrophil apoptosis, as indicated by significantly diminished and delayed PS externalization and nuclear condensation, sustained expression of anti-apoptosis mediators, and delayed processing and activation of caspases-8, 9 and 3 (27–29, 33, 34). Because PMN apoptosis is a crucial aspect of both bacterial clearance and inflammation regulation, we hypothesized that interactions with, and uptake of, *F. tularensis*-infected PMN (iPMN) by primary human MDMs would be impaired. To test this notion, neutrophils isolated from healthy adult donors were aged in culture or infected with *F. tularensis* for 24 h and then incubated with MDM monolayers prior to Hema-3 staining and light microscopy analysis. The data in **Figures 1A–C** indicate that, in sharp contrast to what we predicted, iPMNs interacted more efficiently with MDMs than their aged, uninfected counterparts, as indicated by quantitation of the percentage of MDMs interacting with neutrophils and the number of PMNs per interacting macrophage.

As our Hema-3 staining data suggested that in most instances iPMNs were internalized rather than merely tethered to MDM surfaces (**Figure 1A**), we utilized differential staining to interrogate this further. Specifically, aged or infected PMNs were added to MDM monolayers at a ratio of 5 neutrophils per MDM and then incubated at 37°C for 2h to allow for binding and engulfment. Subsequently, cell mixtures were fixed but not permeabilized, and antibodies to the PMN surface marker CD15 were added to identify neutrophils that were bound to MDMs or partially ingested. After permeabilization, anti-MPO antibodies were added to detect all PMNs (surface-associated and ingested). A schematic of our staining regimen is shown in **Supplementary Figure S1**. Confocal images representative of at least three independent experiments are shown in **Figure 1D**, with quantitation in **Figures 1E, F**. These data were independently validated using flow cytometry. In this case, aged and infected neutrophils were pre-labeled with Cell Trace Far Red, washed, and then incubated with MDMs. Live cell mixtures were stained with FITC-conjugated anti-CD15 antibodies to detect surface-exposed PMNs prior to flow cytometry analysis (**Figures 1G, H**). This approach distinguished MDMs not interacting with neutrophils (unassociated, lower left quadrant) from MDMs containing only fully ingested neutrophils (upper left quadrant), and MDMs with surface-tethered neutrophils or both fully ingested and surface-tethered PMNs in the upper right quadrant. The results of all three assays are concordant and demonstrate that when directly compared, primary human macrophages interacted more efficiently with *F. tularensis*-infected neutrophils than their aged, uninfected counterparts.

### 2.2 Mechanism of iPMN uptake by MDMs

**Figure 2:**
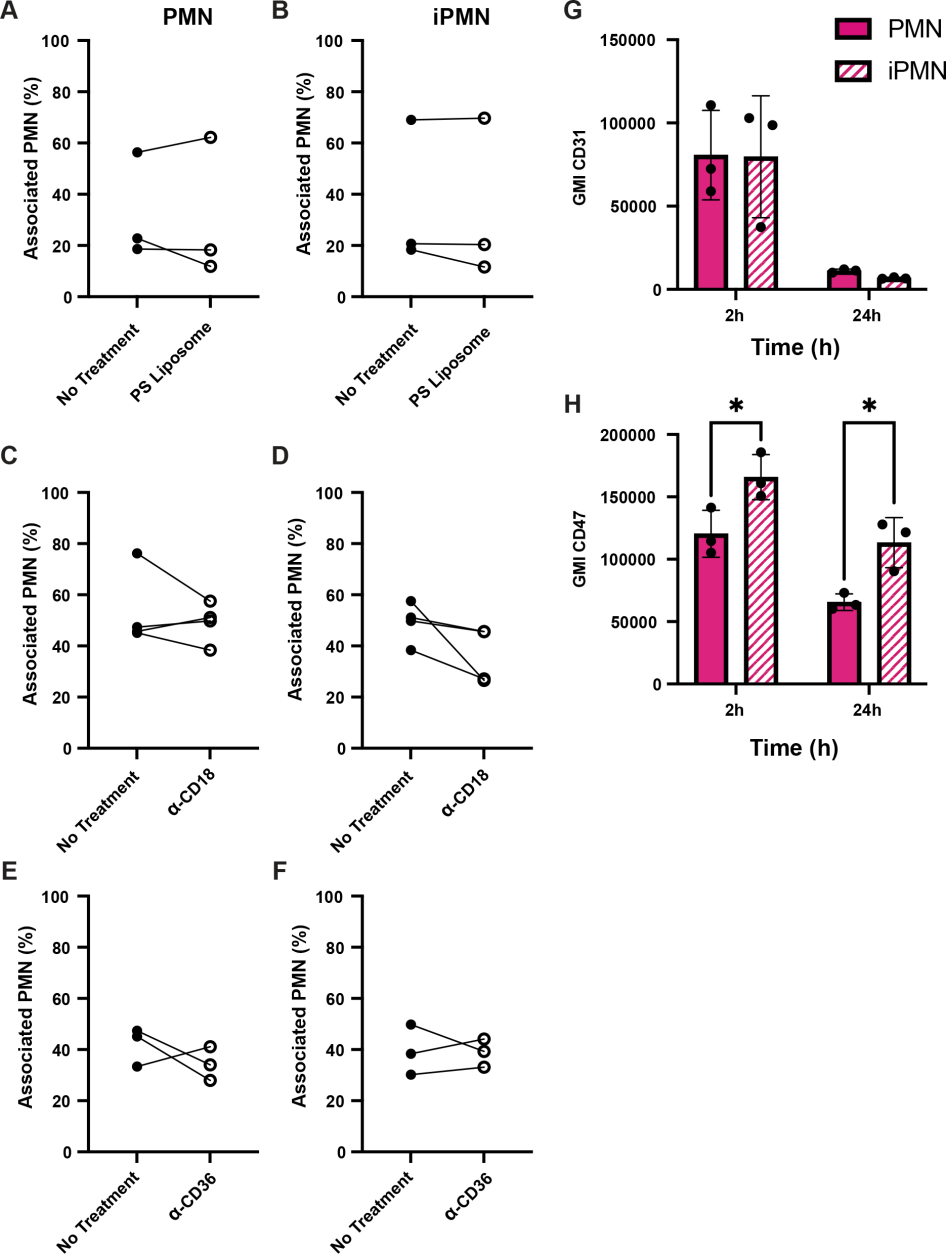
Receptor blocking agents and don’t eat me signal abundance do not account for iPMN uptake. **(A–F)** Neither PS liposomes **(A, B)** nor blocking antibodies to CD18 **(C, D)** or CD36 **(E, F)** impaired interactions of aged (PMN) or infected neutrophils (iPMN) with human macrophages. Results of three independent experiments are shown for each condition. **(G, H)** Flow cytometry quantitation of CD31 **(G)** and CD47 **(H)** on aged and infected neutrophils at 2 and 24 h, as indicated. Geometric mean intensity (GMI) is shown as the average ± SD for three independent experiments. *p<0.05 by Student’s t-test.

Under our experimental conditions, the majority of aged PMNs are PS-positive by 24 h, whereas infected PMNs are not (33) (**Supplementary Figure S2**). As noted above, uptake of aged neutrophils by human macrophages is generally not PS-dependent and as such is not sensitive to inhibition by PS liposomes (6, 7). In keeping with this, we demonstrate here that PS-liposomes had no effect on MDM uptake of aged or infected PMNs (**Figures 2A, B**). Blocking antibodies to the common β2 integrin subunit CD18 and CD36, both of which have also been linked to efferocytosis (7, 8, 35), were also without effect (**Figures C–F**). Next, we interrogated other surface molecules that might promote preferential binding and uptake of iPMN by macrophages. CD31 and CD47 are established “don’t eat me” signals on healthy cells (6, 36, 37). As judged by flow cytometry, surface CD31 was abundant on freshly isolated cells, and declined markedly by 24 h regardless of *F. tularensis* infection (**Figure 2G**). By contrast, CD47 was significantly more abundant on iPMNs at both assayed time points (**Figure 2H**). Other receptors linked to phagocytosis were not differentially expressed (CD32, CD87) or were slightly higher on aged neutrophils (CD35) (**Supplementary Figure S3**). Thus, differences in the abundance of several receptors and don’t eat me signals do not account for preferential interactions between MDMs and *F. tularensis*-infected neutrophils.

### 2.3 Serum C1q is required for infected neutrophil uptake by macrophages

**Figure 3:**
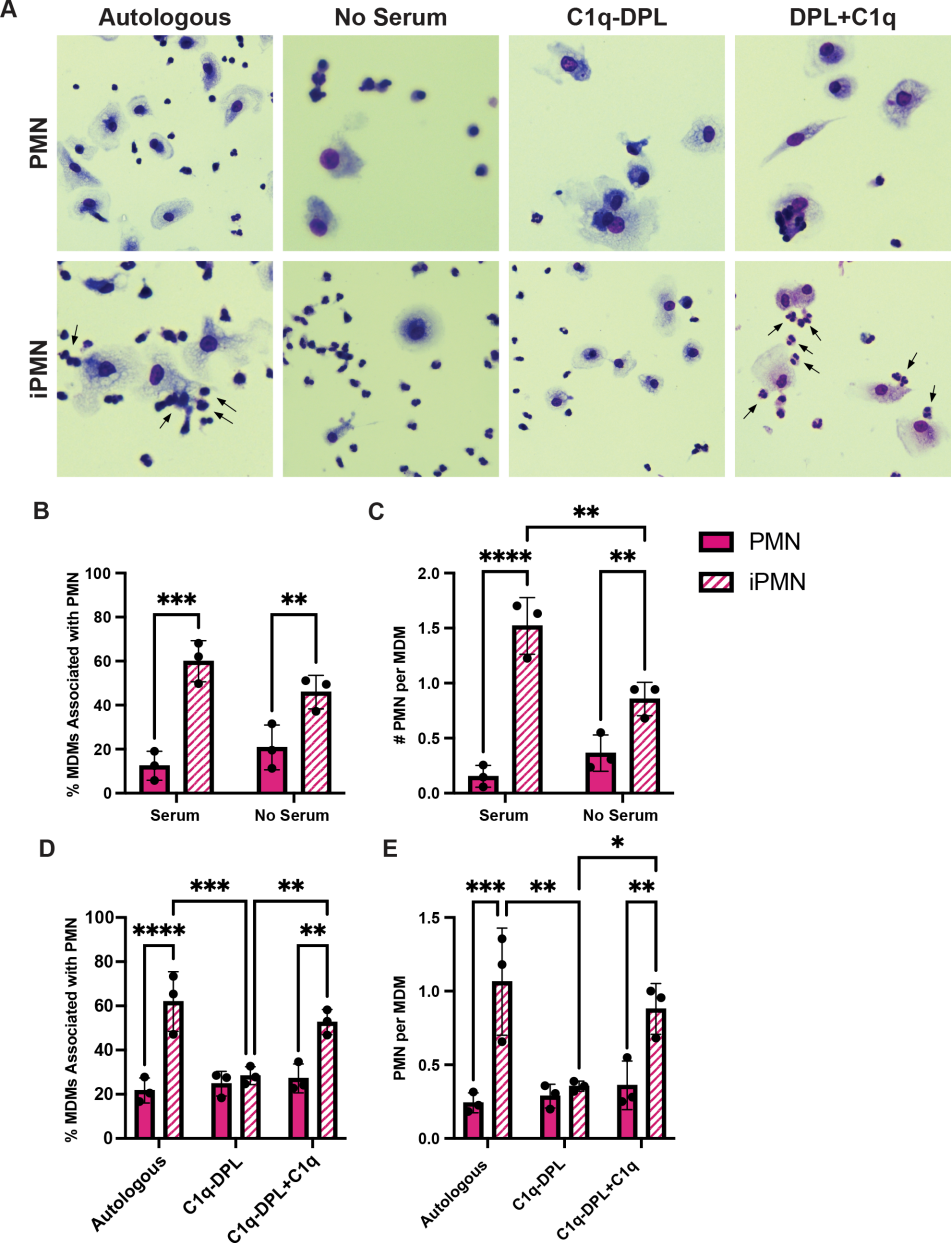
C1q plays a significant role in iPMN uptake. **(A)** Representative Hema-3-strained images of iPMN and PMN cocultured with MDMs in the presence of or absence of autologous donor serum, C1q-DPL serum, or C1q-DPL serum with C1q added back. *Arrows* indicate MDM-iPMN interactions. **(B)** Percentage of MDMs associated with PMN in the presence and absence of autologous donor serum. Data are the mean ± SD of three independent experiments. **p < 0.01, ***p < 0.001 by ANOVA with Tukey’s multiple comparisons post-test. **(C)** Number of PMN associated with each macrophage in the presence and absence of donor serum. Data are the mean ± SD of three independent experiments. **p < 0.01, ****p < 0.0001 by ANOVA with Tukey’s multiple comparisons post-test. **(D)** Percent of total MDMs counted associated with PMN in the presence of autologous donor serum, C1q-DPL serum, or C1q addback to C1q-DPL serum. Data are the mean ± SD of three independent experiments. **p < 0.01, ***p < 0.001, ****p < 0.0001 by two-way ANOVA with Tukey’s multiple comparisons post-test. **(E)** Number of PMN associated with each MDM in autologous donor serum, C1q-DPL serum, or C1q addback to C1q-DPL serum shown as the mean ± SD of three independent experiments. *p < 0.05, **p < 0.01, ***p < 0.001 by two-way ANOVA with Tukey’s multiple comparisons post-test.

The efferocytosis assays used in this study included autologous donor serum. Thus, we interrogated next the role of serum complement in iPMN binding and uptake. First, we tested the serum dependence of these interactions. The representative images (**Figure 3A**) and associated quantitative data demonstrate a significant and selective enhancing effect of fresh serum on the percentage of MDMs that interacted with iPMNs (**Figure 3B**) and the number of iPMNs per MDM (**Figure 3C**). Next, we interrogated the role of specific serum components, focusing on C1q because of its established role as a key regulator of efferocytosis (11, 14, 38). Assays performed in the presence of C1q-depleted serum (C1q-DPL) demonstrated that the absence of this complement component significantly impaired both the percentage of MDMs interacting with iPMNs and the number of iPMNs per macrophage (**Figures 3A, D, E**). A critical role for C1q was validated by adding back recombinant human C1q at 70 μg/mL, as recommended by Complement Technologies, which rescued MDM-iPMN interactions (**Figures 3A, D, E**). By contrast, an absence of serum or C1q specifically did not significantly impair efferocytosis of aged PMNs (**Figures 3A–E**).

**Figure 4:**
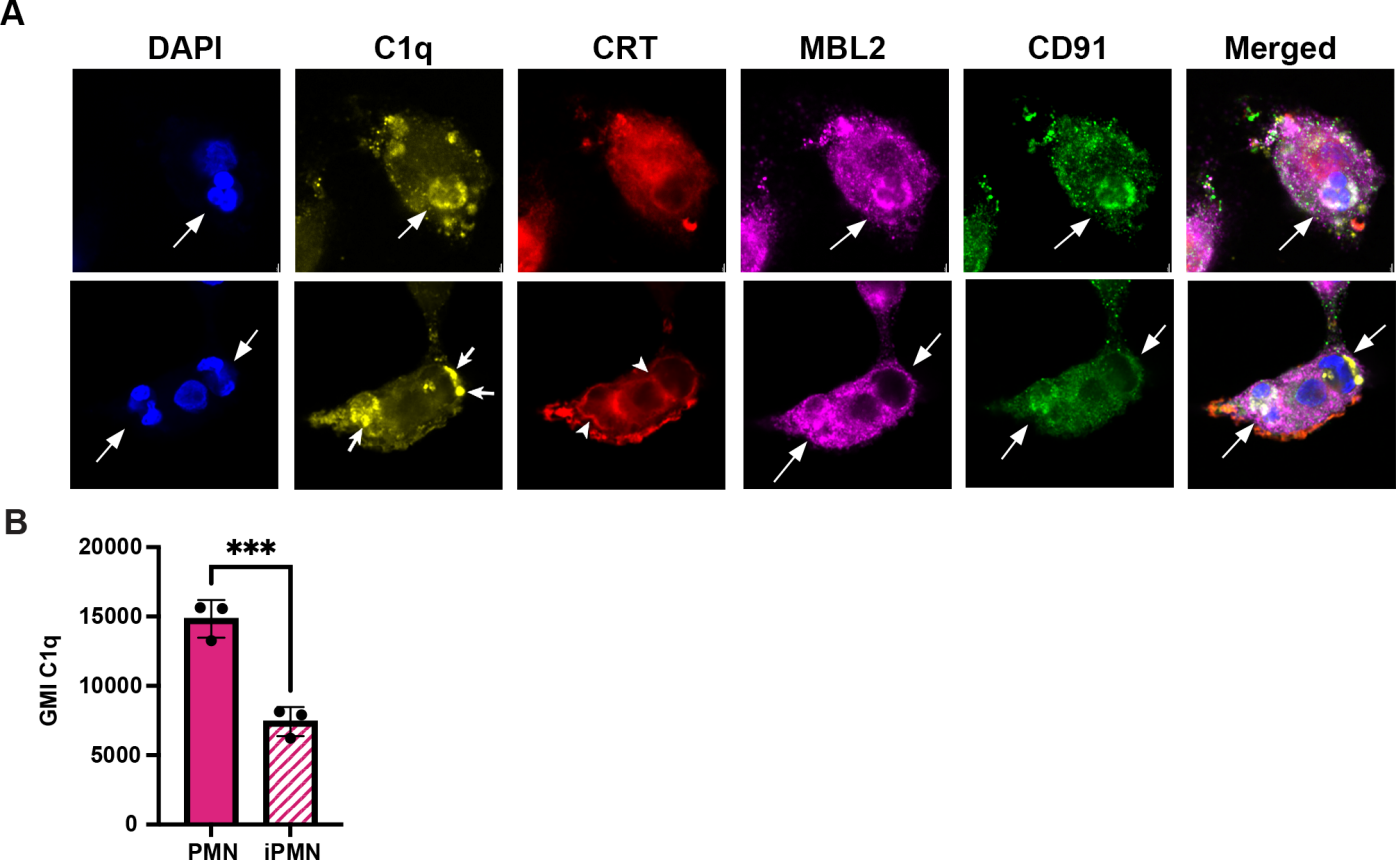
C1q clusters more strongly on iPMNs despite heaver deposition on aged PMNs. **(A)** Representative confocal images of synchronized efferocytosis stained to detect DNA (DAPI, blue) C1q (yellow), calreticulin (CRT, red), mannose-binding lectin (MBL2, purple), and CD91 (green). Arrows indicate efferosomes and specifically mark C1q clusters on iPMNs inside macrophages. Arrowheads note CRT localization at the margin of iPMN efferosomes in MDMs. **(B)** Flow cytometry quantitation of C1q deposition on PMNs shown as geometric mean intensity (GMI) Data are the mean ± SD of three independent experiments. ***p < 0.001 by Student’s t-test.

CD91, also called low density lipoprotein receptor-related protein 1 (LRP1), MBL2, and the “eat me” signal CRT can associate with C1q and aid efferocytosis (11). To determine whether any of these molecules colocalized with C1q at the efferocytic synapse during iPMN uptake, we carried out synchronized efferocytosis assays with confocal microscopy analysis. Specifically, aged or infected PMNs were centrifuged onto MDMs to synchronize binding and then incubated for 20 min at 37°C to allow engulfment prior to fixation, staining, and microscopy. CRT appeared enriched specifically on efferosomes containing iPMNs but neither MBL2 nor CD91 was enriched or excluded from sites were PMNs engaged MDMs (**Figure 4A**).

Importantly, our data also indicate that both aged and infected PMNs ingested by MDMs were C1q-positive but differed in appearance, as C1q was diffusely distributed on aged neutrophils but clustered on iPMNs (**Figure 4A**). To interrogate this further, we used flow cytometry to quantify C1q on PMN surfaces. These data indicate that C1q was more abundant on aged PMNs than on *F. tularensis*-infected PMNs (**Figure 4B**). Thus, we suspect that the surface clustering of C1q may be more important than its abundance as a driver of iPMN-MDM interactions. Taken together, these data reinforce a critical role for C1q, alone or together with CRT, in iPMN uptake.

### 2.4 Infected neutrophils mediate Trojan Horse infection of macrophages

**Figure 5:**
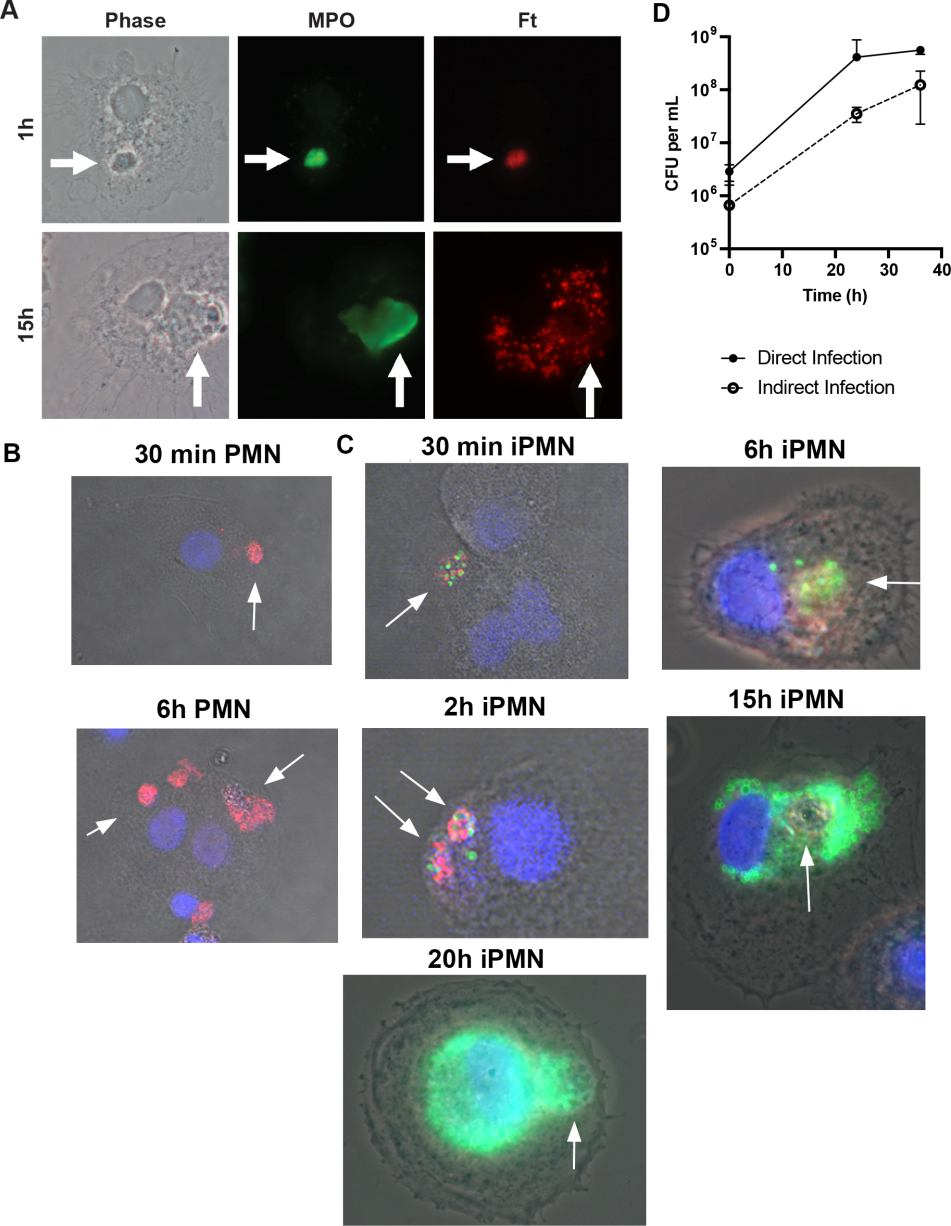
iPMN are Trojan Horses for F tularensis infection of macrophages. **(A)** Representative light microscopy images of iPMNs after 1 h and 15 h coculture with monocyte-derived macrophages. Phase contrast images are shown along with double staining to show MPO in green and *F. tularensis* in red. Arrows indicate PMNs. **(B, C)** Representative confocal images show MDMs at 30 min – 15 h, as indicated, after uptake of aged or infected PMNs. Merged images show DIC along with macrophage nuclei in blue, neutrophil MPO in red and *F. tularensis* in green. **(D)** Kinetics of *F. tularensis* growth in macrophages when delivered indirectly by iPMNs or directly by phagocytosis. Data are the mean ± SD of three independent experiments.

As *F. tularensis* evades killing and escapes the phagosome to replicate in neutrophil cytosol (33), we hypothesized that iPMNs may act as Trojan horses for infection of MDMs by this bacterium. To test this hypothesis, we used immunofluorescence and phase contrast microscopy to assess the fate of ingested neutrophils and their bacterial cargo. At 30–60 min after uptake, phagosomes containing PMN and iPMNs were readily apparent and appeared intact (**Figures 5A–C**). By 2–6 h, *F. tularensis* escaped from enlarged/swollen efferosomes into the cytosol and by 15–24 h had replicated to high density in this locale (**Figure 5C**, **Supplementary Figure S4**). Notably, iPMN remnants and traces of MPO were still apparent inside MDMs 15–24 h after iPMN uptake, suggesting incomplete degradation (**Figure 5C**, **Supplementary Figure 54B**), which contrasts with dispersal and disappearance of MPO and PMN debris from MDMs that ingested aged neutrophils (**Supplementary Figure S4B**).

Quantitation of colony forming units (CFU) demonstrated that *F. tularensis* grew at a similar rate in MDMs when delivered directly by phagocytosis or indirectly via an infected neutrophil (**Figure 5D**). Notably, *F. tularensis* utilizes its T6SS to escape from phagosomes and reach its replicative niche (22, 23). FevR is a virulence regulator that controls expression of genes in the Francisella Pathogenicity Island (FPI) that encode the T6SS (39). In keeping with the critical role of the T6SS, Δ*fevR* mutants are avirulent as they are trapped inside phagosomes and unable to replicate in host cells *in vitro* or animal models of infection (39, 40). However, as they retain expression of bacterial lipoproteins, Δ*fevR* mutants resemble wild-type bacteria in their ability to delay apoptosis and prolong neutrophil lifespan (41). We repeated our efferocytosis assays using neutrophils infected with isogenic Δ*fevR* organisms. Confocal analysis demonstrated that the presence of mutant bacteria inside ingested PMNs 2 h after uptake. At later time points, however, only digested bacterial debris scattered throughout MDMs was apparent (**Supplementary Figure S4**), similar to the dispersal of MPO throughout the cytoplasm of MDMs after ingestion of aged PMNs (**Figures 5B**, **Supplementary Figure S4**). Based on these data, we conclude that virulence genes within the FevR regulon, including the T6SS, are essential for Trojan Horse infection of MDMs.

### 2.5 Infected neutrophils induce a distinct macrophage polarization state

**Figure 6:**
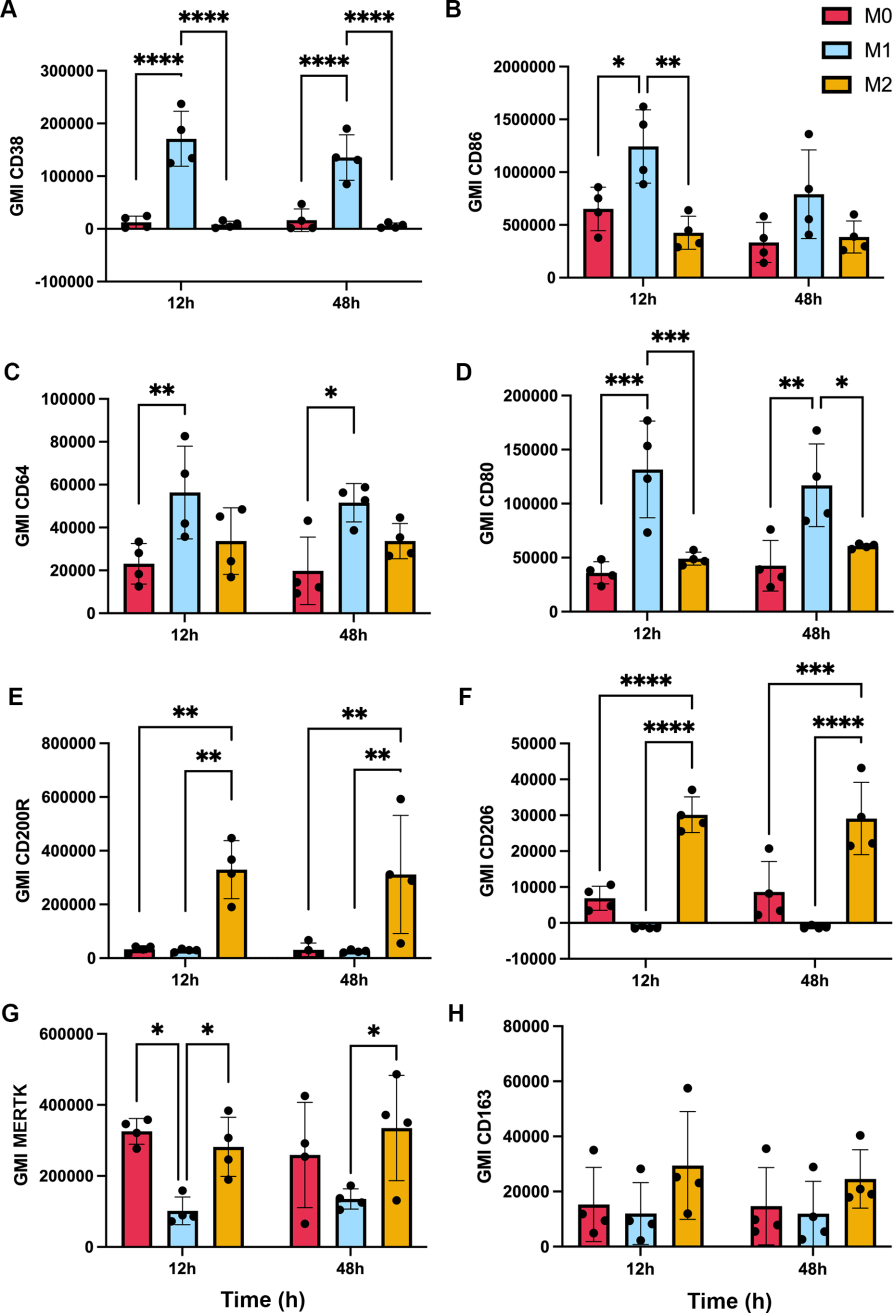
Validation of macrophage polarization. MDMs were left untreated (M0) or were stimulated for up to 48 h with IFNγ + LPS or IL-4 to induce M1 or M2 polarization, respectively, prior to surface marker quantitation by flow cytometry. Graphs show geometric mean intensity (GMI) as the average ± SD from 4 independent experiments. **(A)** CD38. **(B)** CD86. **(C)** CD64. **(D)** CD80. **(E)** CD200R. **(F)** CD206. **(G)** MERTK. **(H)** CD163. *p < 0.05, **p < 0.01, ***p < 0.001, ****p < 0.0001 by ANOVA with Tukey’s multiple comparisons post-test.

**Figure 7:**
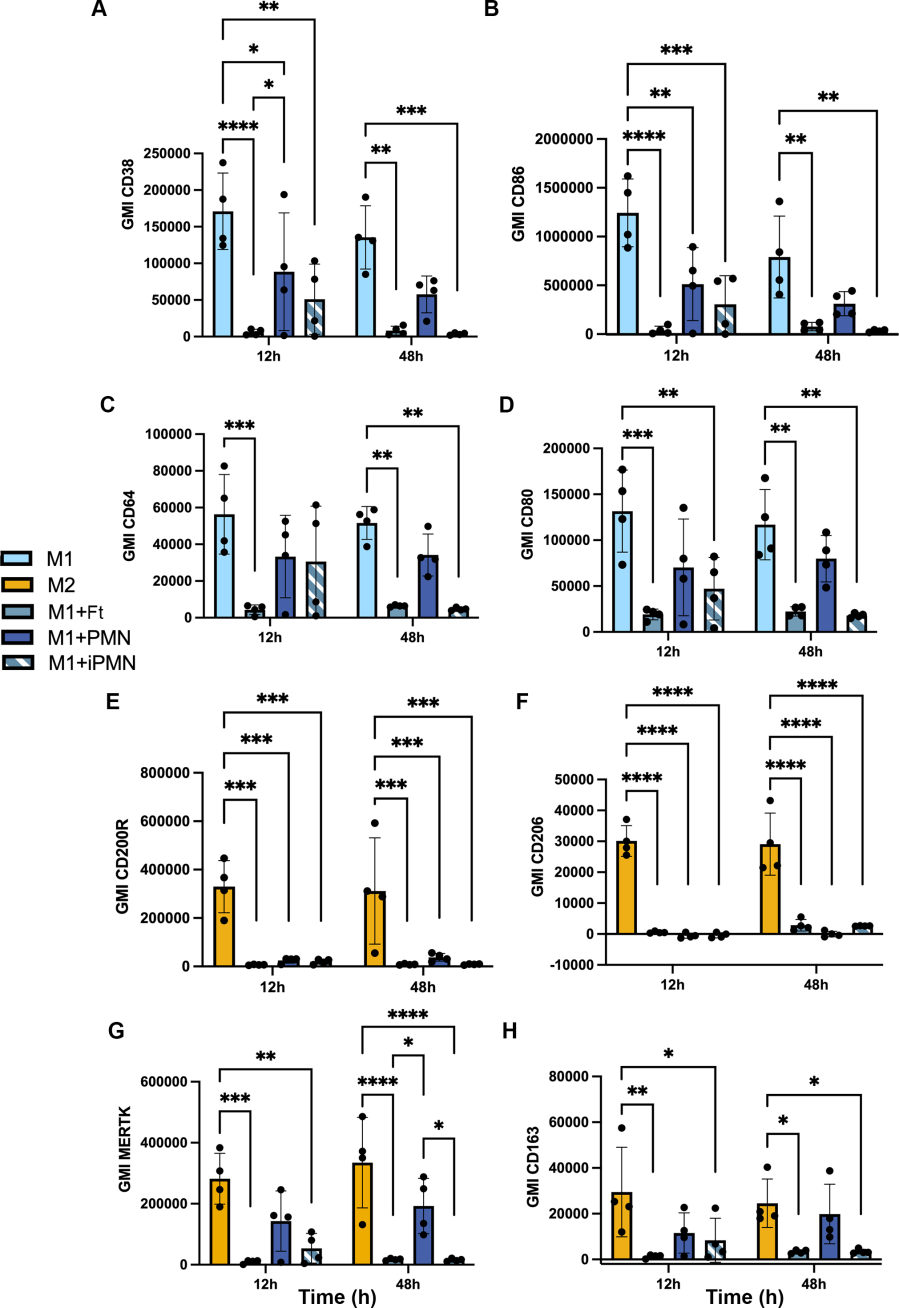
Differential repolarization of M1 macrophages by aged and infected PMNs and direct *F. tularensis* infection. Surface markers of M1 and M2 polarized MDMs and M1 macrophages that ingested *F. tularensis*, aged PMNs or iPMNs were analyzed at 12 and 48 h by flow cytometry. **(A–D)** Geometric mean fluorescent intensity (GMI) of M1 surface markers CD38, CD86, CD64, and CD80, respectively as the mean ± SD (n=4). *p < 0.05, **p < 0.01, ***p < 0.001, ****p < 0.0001 by two-way ANOVA with Tukey’s multiple comparisons post-test. **(E, F)** GMI of M2 surface markers CD200R, CD206, MERTK and CD163, respectively, as the mean ± SD (n=4). *p < 0.05, **p < 0.01, ***p < 0.001, ****p < 0.0001 by two-way ANOVA with Tukey’s multiple comparisons post-test.

The ability of apoptotic cells to reprogram macrophages from a proinflammatory state to a phenotype that favors resolution of inflammation and wound healing has been extensively studied (6, 42), but effects of *F. tularensis*-infected neutrophils on this process are unknown. To address this knowledge gap, we first established conditions for M1 and M2 polarization of MDMs using LPS+IFNγ or IL-4, respectively, and validated the outcomes by flow cytometry analysis of established surface markers (43–50). In each case, unpolarized (untreated, M0) MDMs were analyzed in parallel with their LPS + IFNγ or IL-4-treated counterparts. In our hands, the M1 markers CD38, CD86, CD64 and CD80 all significantly increased on MDMs within 12 h of LPS+IFNγ treatment (**Figures 6A–D**), concordant with published data (46, 51). By contrast, IL-4 significantly increased surface expression of the M2 markers CD200R and CD206, whereas MERTK was abundant on both M0 and IL-4-treated MDMs and CD163 was expressed at low levels under all tested conditions (**Figures 6E–H**). Next, we compared the ability of aged and infected neutrophils to repolarize LPS+IFNγ-treated MDMs and included direct infection with *F. tularensis* as an additional control. We now show that all four M1 surface markers were strongly downregulated by 12 h after direct infection with *F. tularensis*, and similar data were obtained for iPMNs, though downregulation of CD64 was delayed (**Figures 7A–D**). By contrast, aged PMNs elicited significant downregulation of CD36 and CD86, but not CD64 or CD80 (**Figures 7A–D**). Analysis of M2 markers on these same cells showed that CD200R, CD206, CD163 and MERTK remained low on M1 macrophages after incubation with aged PMNs, iPMNs or bacteria alone, whereas MERTK and CD163 increased selectively after incubation with aged PMNs to levels that resembled the IL-4-treated controls (**Figures 7E–H**).

**Figure 8:**
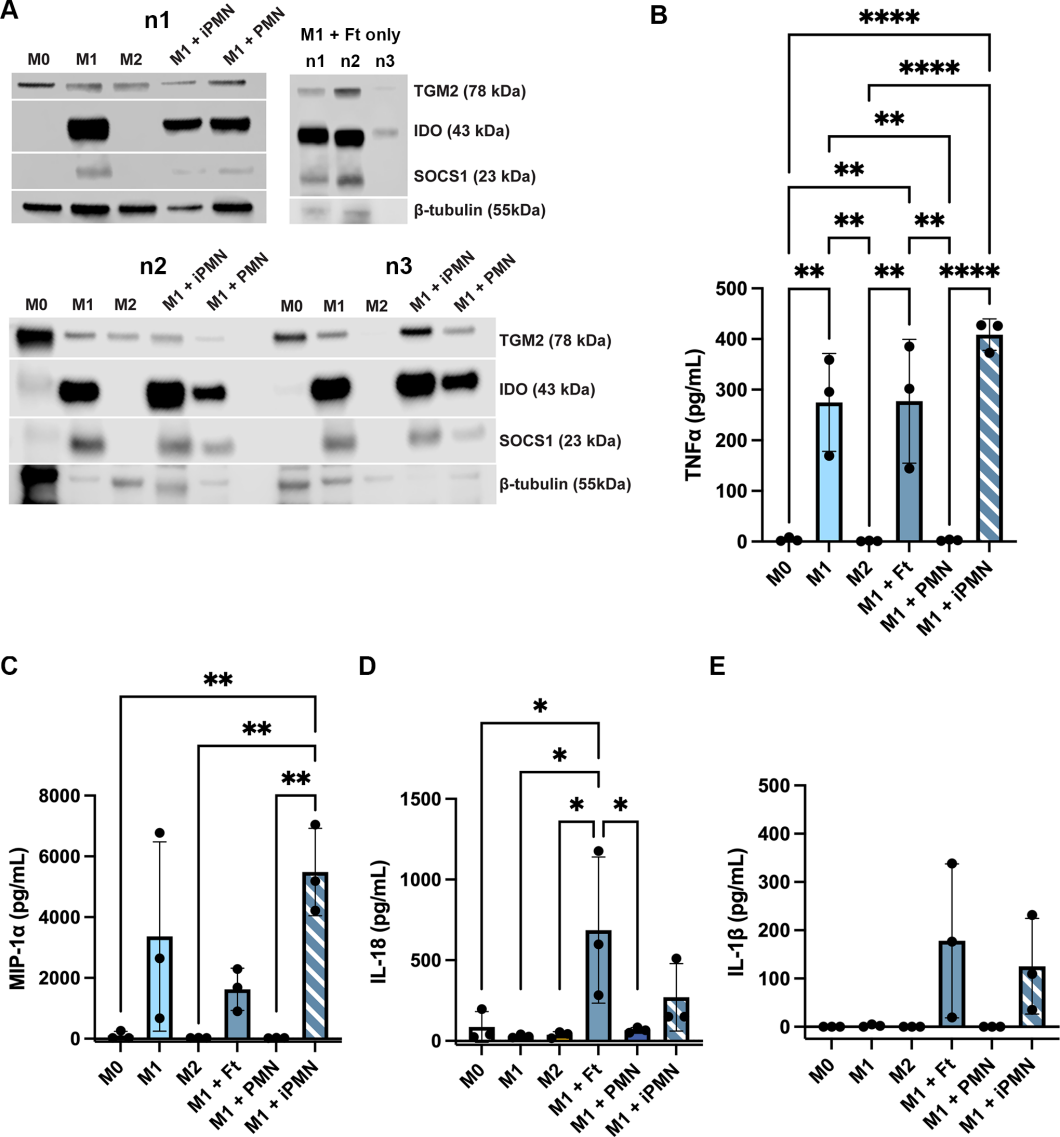
Differential effects of aged and infected PMNs on intracellular M1 markers and cytokine secretion. **(A)** Lysates of M0, M1 and M2 macrophages and M1 macrophages that ingested aged or infected PMNs (upper left and lower left blots) or M1 macrophages directly infected with *F. tularensis* (upper right) were probed to detect TGM2, IDO and SOCS1 with β-tubulin as the loading control. Data from three independent experiments are shown (n1-n3). **(B-E)** Secretion of TNFα **(B)**, MIP-1α **(C)**, IL-18 **(D)** and IL-1β **(E)** were measured by ELISA. Data are the mean ± SD from three independent experiments and were analyzed by two-way ANOVA with Tukey’s multiple comparisons post-test. *p < 0.05, **p < 0.01, ****p < 0.0001.

As the data in **Figure 7** suggest that aged and infected PMNs had distinct effects on MDM polarization, we used western blotting and ELISA to assess additional intracellular markers and secreted cytokines. The data in **Figure 8A** show that indoleamine 2,3-dioxygenase (IDO) and suppressor of cytokine signaling 1 (SOCS1) were abundant in LPS+IFNγ-treated M1 MDMs and cells directly infected with *F. tularensis*, but were at or below the limit of detection in unpolarized and IL-4-treated MDMs and appeared somewhat diminished but not absent after incubation with aged or infected neutrophils. On the other hand, transglutaminase-2 (TGM2) was detected in all but one tested MDM sample. ELISA analysis of secreted cytokines showed that in contrast to aged neutrophils, neither direct *F. tularensis* infection nor uptake of iPMNs significantly altered TNFα or MIP-1α secretion by M1 macrophages (**Figures 8B, C**). Unlike aged neutrophils, *F. tularensis* and iPMNs also stimulated secretion of low levels of IL-18 (**Figure 8D**), whereas trace amounts of IL-1β were not significantly increased over background (**Figure 8E**). Finally, we used dot blot microarrays to profile cytokine secretion by unpolarized MDMs before and after incubation with *F. tularensis*, iPMNs or their aged counterparts and the data in **Supplementary Figure S5** identify a similar pattern as indicated by MIP-1α/MIP-1β secretion under all tested conditions and low levels of TNFα, IL-1β and IL-18 secretion in response to *F. tularensis* or iPMNs. Taken together, our data indicate that iPMNs elicit a complex human macrophage phenotype that supports bacterial growth and persistence.

## 3 Discussion

The goal of this study was to elucidate the fate of *F. tularensis*-infected PMNs. Ultimately, our data demonstrate that iPMN interact more efficiently with MDMs than their aged, uninfected counterparts and that these iPMNs act as Trojan horses, as *F. tularensis* escapes from these compartments to replicate in macrophage cytosol in a FevR-dependent manner with an efficiency similar to direct infection. Trojan horse infection of human or murine macrophages has also been documented for *Chlamydia pneumoniae, Leishmania major, Yersinia pestis, Brucella abortus* and *Mycobacterium tuberculosis* (52–55). For each of these pathogens, iPMN-macrophage interactions were strictly coupled to PS accumulation on dying neutrophil surfaces (52–55). Thus, to our knowledge, uptake of live, infected PMNs by macrophages has not previously been described and may be unique to *F. tularensis*.

In addition to delayed apoptosis, up to 20% of PMNs may lyse within a few hours of *F. tularensis* infection *in vivo*, releasing DNA coated with granule proteins as neutrophil extracellular traps (NETs) (56). We do not observe NETs under our experimental conditions (33, 57). However, we utilize growth conditions that maximize *F. tularensis* virulence factor expression, which may not always be achieved, particularly in complex tissue microenvironments (58, 59). Of note, multiple PMN fates are not without precedent, as accelerated apoptosis that progresses to secondary necrosis, atypical necroptosis and vital NET release have all been reported for neutrophils infected with *Staphylococcus aureus* (60–62).

Although the exact mechanism of iPMN engulfment is yet to be fully understood, we identified fresh serum and C1q as critical to this process. At the same time, our data confirm that C1q is diffusely distributed on apoptotic cells and reinforce evidence that C1q is more abundant on apoptotic cells than their live counterparts (11, 63). Receptors for C1q implicated in efferocytosis include CD35, CD11b/CD18 and CRT (11). We excluded roles for CD35 and CD18 in our model and show that CRT was present at sites of iPMN uptake. CRT is of particular interest as it can bind to cell surfaces in the absence of PS. Additionally, C1q can also interact with MBL2 on apoptotic cells whereas C1q and CRT can interact with CD91 on macrophages (6, 11, 13, 15, 63–65). Plasminogen activator inhibitor-1 can play a role in the engulfment of apoptotic neutrophils by interacting with CRT and vitronectin (66), and CD36, the vitronectin receptor, and thrombospondin have also been implicated in this process (7, 67). Notably, blocking CD36 had no effect in our system and although the other players have not yet been excluded, roles for unknown novel players that may be specific for live PMN uptake should also be considered in future work that is of interest but beyond the scope of the current study.

Canonically, effective defense against infection consists of an early proinflammatory phase that enhances microbe killing and a late phase optimized for wound healing and restoration of homeostasis (68). A variety of cytokines, inflammatory mediators and microbial factors influence macrophage activation state, and it is now appreciated that these cells are highly dynamic and exhibit a spectrum of phenotypes that extends far beyond the original M1/M2 paradigm (44, 69). Adding to this complexity is the fact that fetal bovine serum is more inflammatory than normal human serum and evidence that commonly assayed surface markers are modulated with distinct kinetics (43, 46, 70, 71). Nonetheless, it is unequivocal that uptake of apoptotic cells is a key trigger for termination of inflammation and repolarization of macrophages to a wound healing phenotype *in vivo* and *in vitro* (6, 42, 72).

Direct infection of human monocytes and macrophages with *F. tularensis* actively suppresses pro-inflammatory signaling pathways and diminishes secretion of key proinflammatory cytokines including TNFα and IL-1 by macrophages (73–76). Herein, we sought to develop a deeper understanding of MDM polarization after direct and indirect infection. We differentiated monocytes in normal human serum and validated MDM responses to exogenous IL-4 and INFγ plus LPS. With these data in hand, we demonstrated that direct infection of proinflammatory MDMs with *F. tularensis* elicited an unpolarized surface phenotype, as indicated by marked downregulation of all eight M1 and M2 markers tested, and showed that this was accompanied by upregulation of IDO and SOCS1. IDO and SOCS1 are induced by IFNγ and LPS and are commonly used as M1 markers (77–79). However, their function is anti-inflammatory and immunosuppressive as IDO inhibits T cell proliferation and activation and SOCS1 attenuates cytokine signaling by inhibition of JAK, thereby preventing excessive inflammation (77–79). Thus, it is noteworthy that IDO and SOCS1 remained abundant after uptake of aged and infected PMNs but were at or below the limit of detection in resting and IL-4-treated MDMs. In our hands, aged PMNs stimulated upregulation of MERTK and CD163, markers associated with an M2c wound healing phenotype (80) whereas iPMNs resembled *F. tularensis* in their ability to stimulate downregulation of both M1 and M2 surface markers, albeit with slower kinetics. Overall, these data are consistent with the stealth strategy of *F. tularensis*, limiting its detection and curtailing activation of potentially protective inflammatory responses and defense mechanisms. Whether evasion of TLR4 and upregulation of miR-155 or induction of IDO and SOCS1 play greater roles in limiting cytokine secretion in this model remains to be determined.

In summary, the results of this study demonstrate that *F. tularensis* can use live human neutrophils as vehicles for Trojan horse infection of macrophages and as such identify another potential mechanism for spread of this bacterium throughout a host. Trojan horse infection requires C1q, supports bacterial replication to high density in MDM cytosol and elicits an atypical macrophage phenotype characterized by downregulation of M1 and M2 surface markers concomitant with upregulation of immunosuppressive IDO and SOCS1. As the effects of aged/apoptotic neutrophils were distinct from those of iPMNs and direct *F. tularensis* infection, it will be of interest in future work to determine the extent to which macrophage polarization is driven by signals from replicating bacteria rather than receptors engaged during phagocytosis.

## 4 Materials and methods

### 4.1 Cultivation of bacteria

*Francisella tularensis* subspecies *holarctica* Live Vaccine Strain (LVS) and the isogenic Δ*fevR* mutant have been described (24, 33, 40, 41). Bacteria were grown on Difco cystine heart agar (BD Biosciences, Franklin Lakes, NJ) supplemented with 9% defibrinated sheep’s blood (CHAB) (Hemostat Laboratories, Dixon, CA) in a humidified, 37°C incubator with 5% CO_2_ in air for 48 hours. Bacteria from CHAB plates were collected into 1 mL PBS without divalent cations (Corning, Corning, NY) supplemented with 1 mM D-glucose (Sigma-Aldrich, St. Louis, MO) and washed twice by centrifugation. Bacteria were quantified by measurement of absorbance at 600nm.

### 4.2 Ethics statement

Heparinized venous blood was acquired from healthy adult volunteers who provided written informed consent in accordance with protocols approved by the Institutional Review Board for Human Subjects at the University of Missouri (#2031144 and #2033122) and the University of Iowa (#201609850 and #200307026).

### 4.3 Isolation of neutrophils and monocytes from human blood and macrophage differentiation

Neutrophils in heparinized, venous blood from healthy adult donors were isolated using established procedures (81). Briefly, the majority of erythrocytes were sedimented from whole blood using 3% dextran (Pharmacosmos, Holbæk, Denmark). Next, neutrophils were separated from mononuclear cells by centrifugation through Ficoll-Hypaque gradients (Cytiva, Chicago, IL). Finally, the neutrophil layer was collected, residual erythrocytes were removed by brief hypotonic lysis, and the isolated neutrophils were resuspended at 5 x 10^6^ cells/mL in serum-free HEPES-buffered RPMI-1640 medium (Gibco, Grand Island, NY) supplemented with 2 mM L-Glutamine. This method routinely yields >95% neutrophil purity. Additionally, the peripheral blood mononuclear cell (PBMC) layer from the Ficoll-Hypaque separation was collected, washed in RPMI-1640 medium, and monocytes were purified using a StemCell Technologies (Vancouver, Canada) Monocyte Enrichment Kit according to the manufacturer’s instructions. Monocytes were counted on a hemacytometer and seeded at 2x10^6^ cells/mL in serum-free HEPES-buffered RPMI-1640 medium with L-glutamine and 20% autologous donor serum in screw-capped Teflon jars and then cultured at 37°C in 5% CO_2_ for 5–7 days for differentiation into MDMs, with feeding on day 4 (21, 82, 83). Replicate experiments utilized macrophages and neutrophils from different donors.

### 4.4 Efferocytosis of neutrophils and phagocytosis of bacteria

Freshly isolated human PMNs were aliquoted into 14mL sterile, pyrogen-free polypropylene round bottom, snap-capped tubes and then incubated in suspension for 24 h at 37°C in the presence or absence of *F. tularensis* at a multiplicity of infection (MOI) of 200:1. MDMs were removed from Teflon jars, rinsed twice by centrifugation and then plated into 8-well chamber slides (Corning, Corning, NY) or UpCell 6-well cell culture plates (Thermofisher Scientific Nunc, Rochester, NY) at 2x10^6^ MDM/mL in HEPES-buffered RPMI-1640 supplemented with L-glutamine and 20% autologous donor serum (complete medium) for microscopy or flow cytometry, respectively. After 24 h of aging or infection, PMNs were collected, washed three times via centrifugation to remove extracellular bacteria or debris, counted, and added to washed MDM monolayers at a ratio of 5 PMN per MDM in complete medium and incubated for 2h at 37°C unless otherwise stated.

### 4.5 Hema-3 staining and light microscopy

Chamber slide wells were rinsed three times to remove unattached PMNs. Thereafter, cell monolayers were stained with PROTOCOL Hema-3 reagents (Fisher Scientific, Hampton, NH) according to the manufacturer’s instructions. Cells were analyzed using a Zeiss Axioplan 2 light microscope (Carl Zeiss, Inc. Thornwood, NY) or a Leica DMi8 light microscope and LASX software v. 3.7.4.23463 (Leica Microsystems, Buffalo Grove, IL). Representative images were acquired using a 63x or 100x objective. The total number of neutrophils interacting with or ingested by a minimum of 100 MDMs were counted in random fields of view in each of three chamber slide wells for each condition and experiment.

### 4.6 Quantitation of efferocytosis

#### 4.6.1 Confocal microscopy

At the end of the incubation period, chamber slide wells were rinsed to remove unattached cells and monolayers were fixed by incubation for 15 min in fresh 2% paraformaldehyde (PFA) in PBS and then blocked 1 h at room temperature in microscopy blocking buffer (PBS supplemented with 5 mg/mL BSA, 0.5 mg/mL NaN_3_, and 10% horse serum, all from Sigma-Aldrich). Thereafter, cells were stained with polyclonal anti-human CD15 antibodies (Cat. No. BS-1702R, Cell Signaling Technology, Danvers, MA), diluted 1:100 in blocking buffer, for 1 h at room temperature and then rinsed six times in PAB (PBS supplemented with 5 mg/mL BSA and 0.5 mg/mL NaN_3_). Next, cells were permeabilized with ice-cold 1:1 methanol:acetone for 5 min and then rinsed with PBS, followed by staining with 1:100 dilution of anti-human MPO antibodies (Cat. No. MAB3174, 392105, Cell Signaling Technology, Danvers, MA) for 1 h at room temperature and then rinsed. Affinity purified F(ab’)_2_ secondary antibodies conjugated to Alexa Fluor(AF)488 or AF647 (Jackson ImmunoResearch, West Grove, PA) were used at 1:200 dilution. After 1 h at room temperature, wells were rinsed with PAB, chambers were removed, and coverslips were mounted to each slide in Prolong Diamond mountant with DAPI (Invitrogen, Eugene, OR). Stained cells were analyzed via confocal microscopy using a Zeiss LSM510 with Zen Software (Carl Zeiss, Inc.) or a Leica Stellaris 5 microscope and LASX software 4.5.0.25531 (Leica Microsystems). In each case, MDM-associated neutrophils were counted and scored with MPO staining used to detect all neutrophils and CD15-staining used to detect PMNs that were surface associated or incompletely ingested. Once again, at least100 MDMs were counted per well for three wells/condition in each experiment.

#### 4.6.2 Flow cytometry

Aged or infected neutrophils at 8x10^6^/mL were pre-labeled with 1 μM CellTrace Far Red (Invitrogen) diluted in PBS for 30 min at 37°C, washed twice with PBS, resuspended in medium, and added to MDM monolayers in UpCell dishes or chamber slides at a ratio of 5 or 20 neutrophils/MDM followed by incubation for 30–120 min at 37°C. Thereafter, non-adherent cells were washed away using warm RPMI-1640 medium and remaining cells were harvested by trypsinization. Collected cells were washed with medium and then stained with phycoerythrin (PE)-conjugated anti-CD15 antibodies (Cat. No. IM1954U, Beckman Coulter) for 30 min on ice, washed three times with FACS buffer (RPMI-1640 supplemented with 10% heat-inactivated FBS), and then analyzed on an Accuri C6 flow cytometer (BD Biosciences, Sparks, MD). Each data set was first gated on macrophages as determined by forward and side scatter and then subdivided into three distinct populations according to their CellTrace and CD15-PE fluorescence intensities. Three thousand events were collected for each sample and the data were analyzed using Accuri C6 software.

### 4.7 Efferosome markers

#### 4.7.1 Flow cytometry of neutrophil surface markers

Aged and infected PMNs were collected, adjusted to 1x10^6^ in 100 mL total volume FACS buffer (PBS-/- with 2% FBS) and then stained with 5 μl each of: anti-CD31-PE, anti-CD47-APC, anti-CD32-PerCP/Cy5.5, anti-CD87-PE (all from BioLegend, San Diego, CA) or anti-CD35-PE (from BD Biosciences, Franklin Lakes, NJ) on ice for 20 min. After quenching with 1 mL 1:1 FBS/PBS and washing with PBS by centrifugation, cells were resuspended in 400 μL PBS and run on a CytoFlex V5B5R3 (Beckman Coulter, Brea, CA). Debris was excluded by FSC-H/SSC-H gating followed by doublet exclusion with SSC-A/SSC-H gating. In each case, a minimum of 10,000 events per sample were acquired in the single cell gate. Data were analyzed using FlowJo V.10 software.

#### 4.7.2 C1q microscopy

As indicated, additional efferocytosis experiments were performed using C1q-deficient human or deficient serum reconstituted with 70ug/mL of recombinant human C1q (both from Complement Technology, Inc., Tyler, TX). Thereafter, samples were stained with Hema-3 reagents and analyzed as described above or were fixed with 4% PFA and blocked as described above and then stained with primary antibodies: 1:50 anti-C1q(3R9/2) (Cat. No. MA1-83963), 1:50 anti-mannose-binding lectin-2 (MBL2) (Cat. No. PA5-106674, polyclonal), 1:50 anti-calreticulin (Cat. No. PA1-902A, polyclonal) (all from Invitrogen), 1:25 anti-CD91 (also called LRP-1 Cluster II (Cat. No. AF2368, polyclonal, Biotechne, Minneapolis, MN). Secondary antibody staining was performed as described above. Cells were analyzed using a Leica Stellaris 5 confocal microscope as described above.

### 4.8 Trojan horse infection of macrophages

#### 4.8.1 Microscopy

Efferocytosis assays were caried out as described above with analysis at time points 2–24 h later. Processing for immunofluorescence and confocal microscopy were carried out as described above using 1:100 anti-human MPO mAb (R&D Systems, Minneapolis, MN), and 1:5,000 anti-*Francisella tularensis* antiserum (BD, Sparks, MD).

#### 4.8.2 Quantitation of CFU

MDM monolayers were washed to remove non-adherent cells and overlaid with either infected neutrophils (ratio 5:1) or *F. tularensis* (MOI 100:1) in RPMI with 10% serum. Cells were incubated for 2 h at 37°C in 5% CO_2_, then washed three times to remove extracellular bacteria and unbound neutrophils, and fresh RPMI with 10% serum was added. At 0-, 24-, and 36-hpi, 0.5% saponin was added to each well. Lysates were serially diluted in HBSS without divalent cations and aliquots were spotted onto CHAB plates prior to incubation at 37°C as we described (83). Viable bacteria were determined by counting CFU.

### 4.9 Macrophage polarization

After differentiation for 5–7 days, MDMs were left unpolarized (M0) or were treated with 50 ng/mL recombinant human IFNγ (Peprotech, Cranbury, NJ) and 10 ng/mL LPS from *Escherichia coli* 0111:B4 (Invivogen, San Diego, CA) in RPMI-1640 and 20% autologous human serum for 36–48 h for M1-like polarization or 50 ng/mL of recombinant human IL-4 (Peprotech) under the same conditions to achieve M2 polarization. As indicated, PMN, iPMN or *F. tularensis* were added to M1 polarized cells for 2 h to allow for uptake. After rinsing to remove free cells or bacteria, MDMs were incubated for an additional 12 h at 37°C to allow for repolarization prior to analysis.

#### 4.9.1 Flow cytometry analysis of surface markers

Fcγ receptors were blocked using Human BD Fc Block (BD Pharmingen) for 10 min at room temperature according to manufacturer instructions. Cells at 1 x10^6^/mL were stained for flow cytometry using 5 μl of each antibody in FACS buffer (2% FBS in PBS without calcium and magnesium) for 30 min on ice. Flow cytometry antibodies were from BioLegend: FITC anti-CD80 (#305206), PE/Cy7 anti-CD86 (#305421), PE/Dazzle anti-CD200R (#329309), BV510 anti-CD38 (#356611), PerCP anti-CD163 (#333625), PE anti-MERτκ (#367607), AF700 anti-CD206 (#321131), BV750 anti-HLA-DR (#307671), and BV510 anti-CD64 (#305027). Samples were run on a Beckman Coulter Cytoflex R5-V5-B3. Doublets were gated out using FSC/SSC parameters and PMNs were excluded using Pacific Blue anti-CD66b (#305111). FlowJo V10.7.1 software was used for data analysis.

#### 4.9.2 Western blotting

Cell lysates were prepared as described (30, 33) and total protein was quantified using Pierce BCA Protein Assay Kits (Thermo Fisher Scientific) according to manufacturer instructions. Proteins in each lysate were separated on NuPAGE 10% Bis-Tris Gels (Invitrogen, Carlsbad, CA) using 20 μg total protein per lane. Blocked membranes were probed with a 1:1,000 dilution each of anti-TGM2 (D11A6, Cat. No. 3557T), anti-IDO (D5J4E Cat. No. 86630T) and anti-SOCS1 (E4K7Q, Cat. No. 68631T), all from Cell Signaling Technology. Blots were then stripped and re-probed with 1:1,000 dilution of anti-β-tubulin (T5201, Millipore Sigma). Horseradish peroxidase-conjugated secondary antibodies (Cell Signaling Technology, Cat. No. 7076S and 7074S) were used at 1:2,000. Band detection was completed using Super Signal West Femto Maximum Sensitivity Substrate (Thermo Fisher Scientific) and imaged using the Li-Cor Odyssey XF and Image Studio Software.

#### 4.9.3 Quantitation of cytokine secretion by ELISA

Supernatants were collected from MDM monolayers and clarified by centrifugation. Cytokines were quantified using the following ELISA kits according to manufacturer instructions: Human CCL3/MIP-1α ELISA Kit (R&D Systems, #DMA00), Human TNFα uncoated ELISA Kit (Invitrogen, #88-7346-88), Human IL-18 ELISA Kit (R&D Systems, # BMS267-2TEN), Human IL-1β ELISA Kit (Invitrogen, # KHC0011).

#### 4.9.4 Dot blots

MDMs were left unpolarized and untreated, were directly infected with *F. tularensis*, or were allowed to ingest aged or infected PMNs for 2 h at which time MDM monolayers were washed and then incubated for 12 h at 37°C prior to collection of supernatant medium. Each supernatant was clarified by centrifugation and then used to probe Proteome Profiler Human Cytokine Array Kit A slides (R&D Systems) according to the manufacturer’s instructions. Densitometry was calculated using Fiji Image J, and the heat map was generated using the pheatmap package in R.

### 4.10 Detection of surface PS

Equal numbers of aged and infected PMNs were mixed and then stained with Annexin V-FITC (Invitrogen) to detect surface-exposed PS as we described (30, 33). After fixation and permeabilization, cells were stained with anti-*F. tularensis* antiserum and secondary F(ab’)_2_ antibodies conjugated to TRITC (Jackson ImmunoResearch) (33) and images were obtained using a Zeiss Axioplan 2 as described above.

### 4.11 Statistical analysis

All data are plotted as mean ± standard deviation (SD) from at least three independent experiments using cells from different donors. Data were analyzed using GraphPad Prism version 10 software with *p* < 0.05 indicating statistical significance. Experiments with one control and one experimental group were analyzed using Student’s t-test. Data from experiments with multiple variables were analyzed using ANOVA with Tukey’s multi-comparisons posttest. Details are provided in each figure legend.

## Data Availability

The original contributions presented in the study are included in the article/**Supplementary Material**. Further inquiries can be directed to the corresponding author.
