# Supplementary material for: Francisella tularensis-infected human neutrophils are trojan horses for infection of macrophages
Source: Front Immunol. 2025 Sep 4;16:1632942. doi: 10.3389/fimmu.2025.1632942 (PMC12443744; doi:10.3389/fimmu.2025.1632942)
Supplement: Supplementary file 1 [file DataSheet1.pdf]

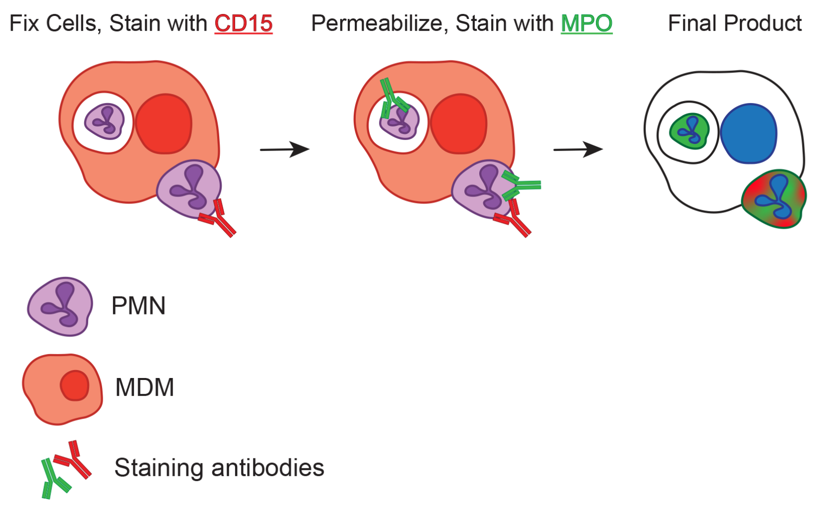

SUPPLEMENTAL FIGURE S1. Schematic diagram of differential staining used to distinguish fully engulfed and surface-exposed PMNs by confocal microscopy. Surface-exposed neutrophils were detected by staining fixed cell monolayers with antibodies to the PMN surface marker CD15. After permeabilization, all neutrophils (surface-exposed and fully ingested by macrophages) were detected using antibodies to MPO.

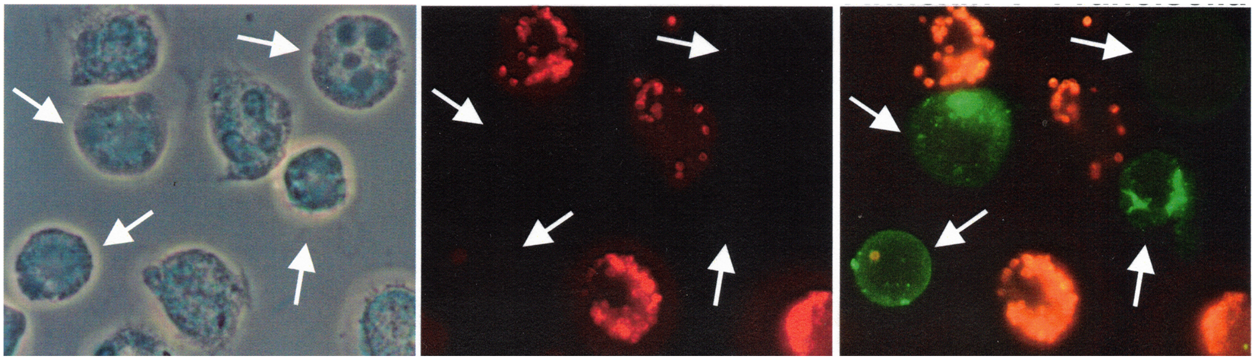

SUPPLEMENTAL FIGURE S2. Infected PMNs do not bind Annexin V-FITC. Light microscopy images of aged and infected PMN mixtures stained to detect exposed PS using Annexin V-FITC (green) followed by permeabilization and staining to detect *F. tularensis* (red). Phase contrast, red fluorescence and combined red and green fluorescence images are shown. Note that all four infected cells are Annexin V-negative, whereas 3 of 4 uninfected PMNs (*arrows*) are Annexin V-positive.

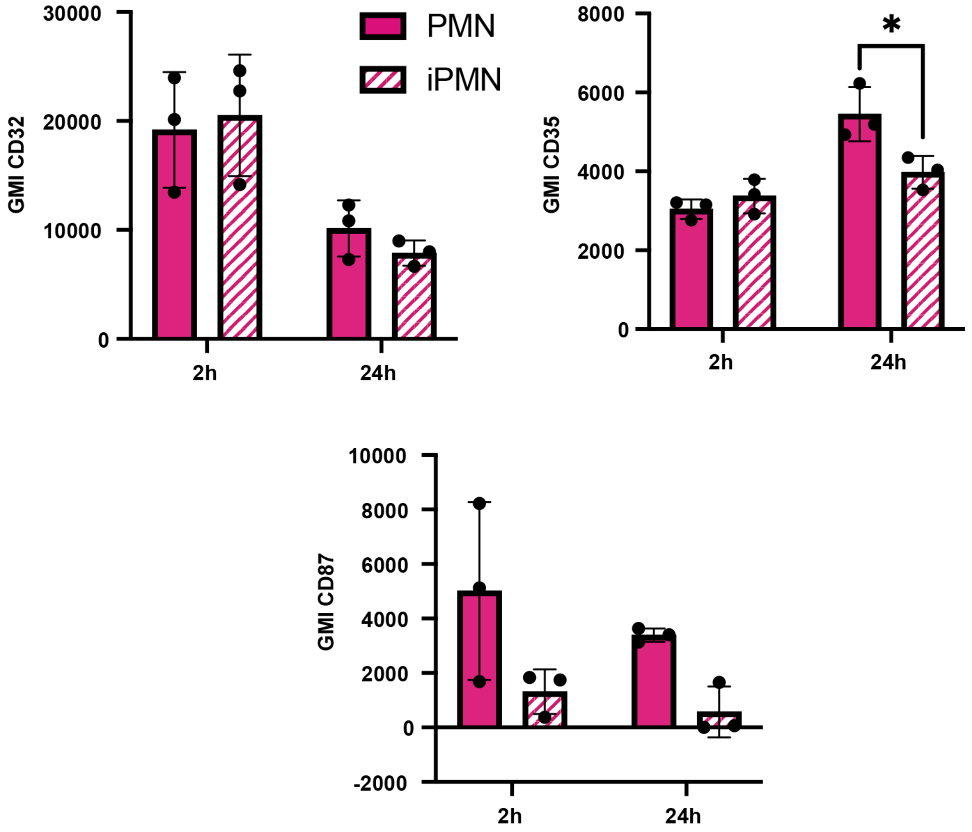

SUPPLEMENTAL FIGURE S3. Quantitation of phagocytosis receptors. Flow cytometry quantitation of CD32, CD35 and CD87 on aged and infected neutrophils at 2 and 24 h, as indicated. Geometric mean intensity (GMI) at each time point is shown as the mean ± SD of three independent experiments. *p<0.05 by Students t-test.

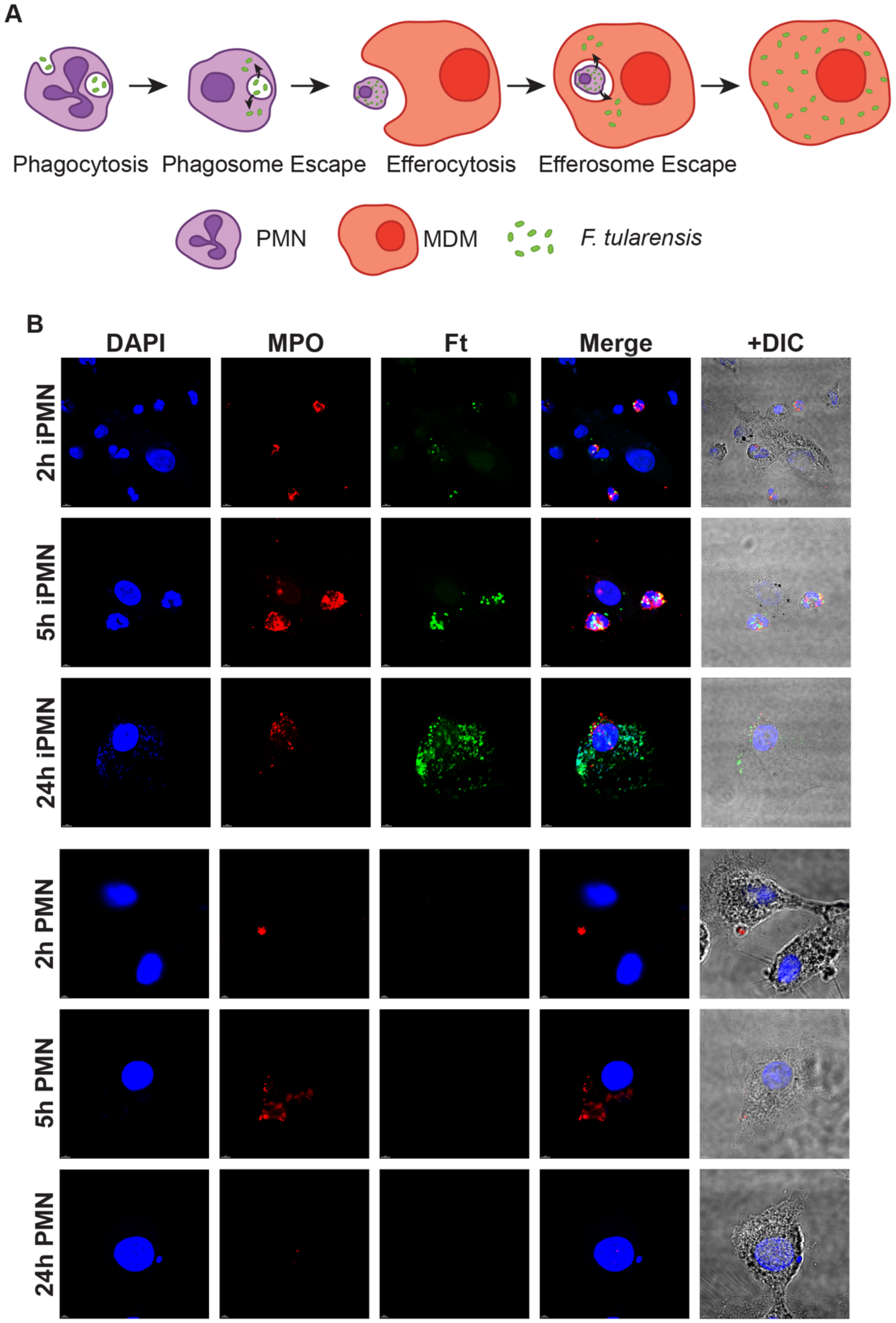
A
Phagocytosis
Phagosome Escape
Efferocytosis
Efferosome Escape
PMN
MDM
F. tularensis
B
DAPI
MPO
Ft
Merge
+DIC
2h iPMN
5h iPMN
24h iPMN
2h PMN
5h PMN
24h PMN

SUPPLEMENTAL FIGURE S4. Time course of Trojan horse infection and aged PMN degradation in MDMs. (A) Schematic diagram depicting delivery of *F. tularensis* into macrophages by infected PMNs (iPMNs). **(B)** Representative confocal images at 2, 5, and 24 h of iPMN and PMN cocultured with macrophages. Fixed and permeabilized cells were stained to show MPO in red and *F. tularensis* (Ft)in green. DNA was detected with DAPI (blue).

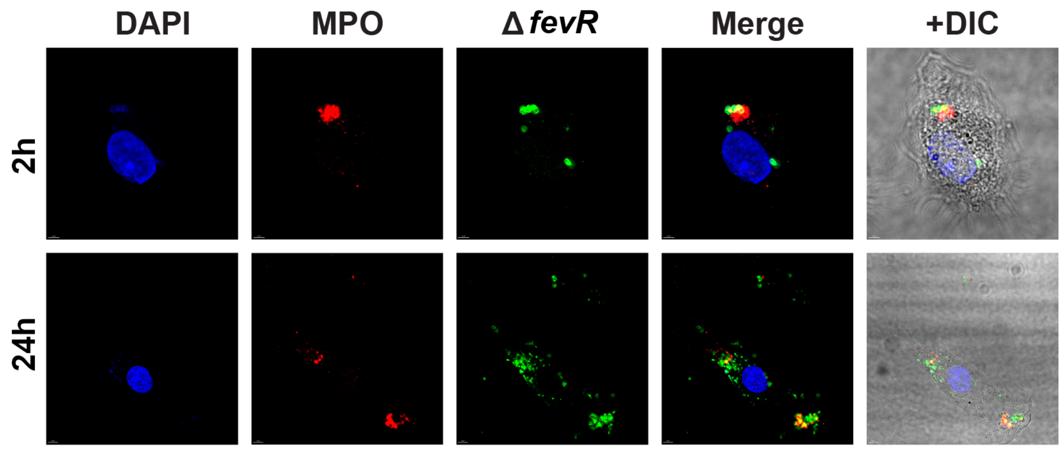

SUPPLEMENTAL FIGURE S5. Δ*fevR* mutants are degraded after delivery into macrophages by iPMNs. Representative confocal images show Δ*fevR F. tularensis* inside neutrophils 2 h after uptake by macrophages and dispersal of degraded bacterial fragments in macrophages at 24 h. Cells were stained to show MPO in red and bacteria in green. DNA was detected with DAPI (blue). DIC, differential interference contrast.

A

	1,2	3,4	5,6	7,8	9,10	11,12	13,14	15,16	17,18	19,20
A	Ref.	CCL1	CCL2	MIP-1	CCL5	CD40L	C5/C5a	CXCL1	CXCL10	Ref.
B		CXCL11	CXCL12	G-CSF	GM-CSF	CD54	IFN-γ	IL-1α	IL-1β	
C		IL-1ra	IL-2	IL-4	IL-5	IL-6	IL-8	IL-10	IL-12	
D		IL-13	IL-16	IL-17A	IL-17E	IL-18	IL-21	IL-27	IL-32α	
E	Ref.	MIF	PAI-1	TNF-α	TREM-1					Neg. Ctl

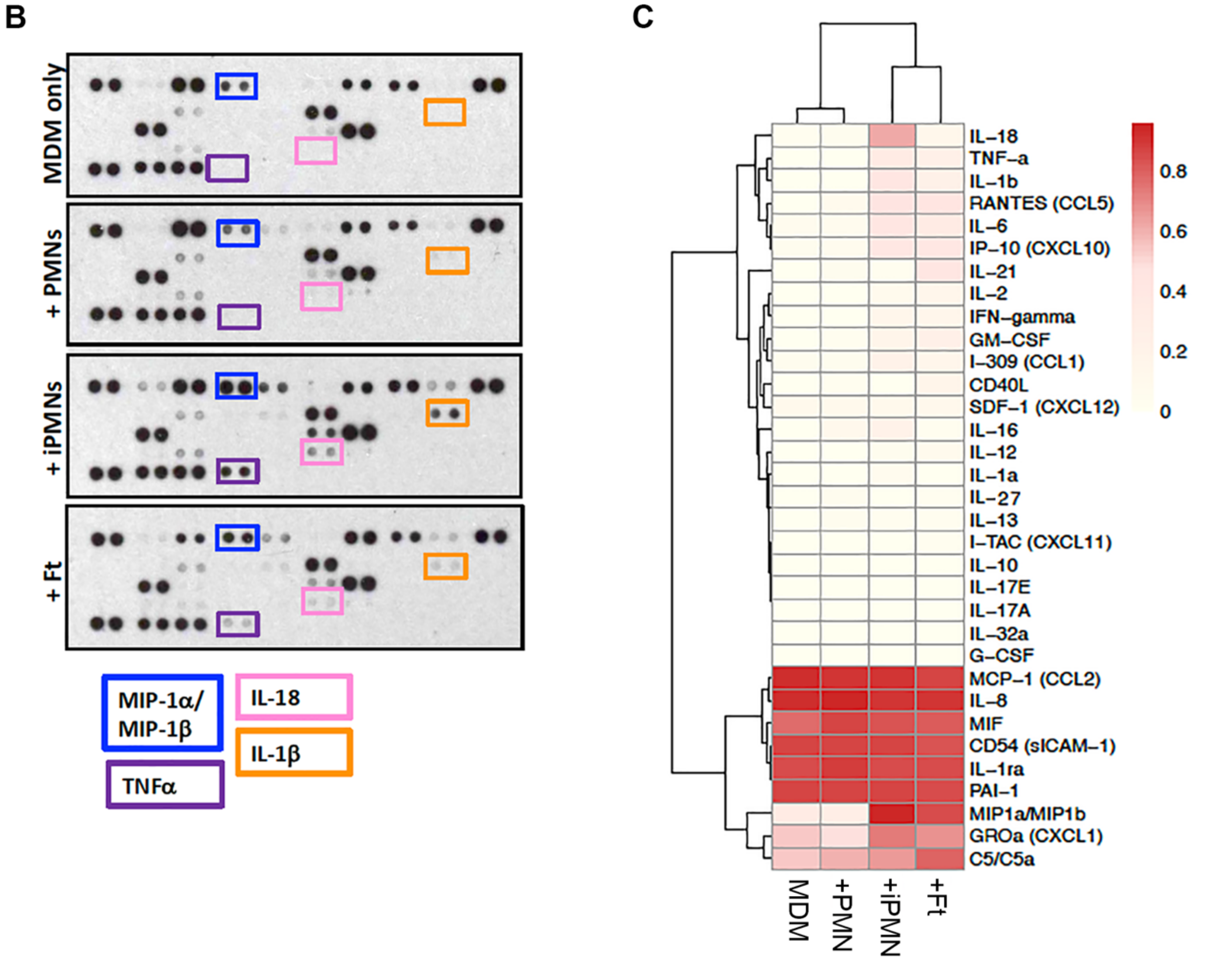

SUPPLEMENTAL FIGURE S6. Cytokine secretion by unpolarized macrophages. Supernatant media from unpolarized monocyte-derived macrophages (MDMs), MDMs directly infected with *F. tularensis* (Ft) for 24 h, or medium from MDMs 24 h after uptake of aged PMNs or infected neutrophils (iPMNs) was used to probe dot blot slide arrays. **(A)** Schematic of dot blot slide array. **(B)** Representative data from one of four independent experiments. **(C)** Pooled data from four experiments displayed as a heat map.
